# The Application of Thermal Solar Energy to High Temperature Processes: Case Study of the Synthesis of Alumina from Boehmite

**DOI:** 10.1155/2014/825745

**Published:** 2014-01-09

**Authors:** Isabel Padilla, Aurora López-Delgado, Sol López-Andrés, Marta Álvarez, Roberto Galindo, Alfonso J. Vazquez-Vaamonde

**Affiliations:** ^1^Centro Nacional de Investigaciones Metalúrgicas (CENIM-CSIC), Avenida Gregorio del Amo 8, 28040 Madrid, Spain; ^2^Departamento de Cristalografía y Mineralogía, Facultad de Ciencias Geológicas, UCM, C/Antonio Nováis, 28040 Madrid, Spain

## Abstract

The aim of this paper is to evaluate the feasibility of obtaining alumina from boehmite using a free, clean, and unlimited power source as the solar energy. Boehmite was obtained by hydrothermal treatment of a hazardous waste coming from aluminum slag milling. The waste is considered as a hazardous substance because of it releasing toxic gases (hydrogen, ammonia, methane, and hydrogen sulfide) in the presence of water. The as-obtained boehmite was transformed into alumina, in air atmosphere, using a solar energy concentrator (Fresnel lens). The solar installation provides a power density of 260 W*·*cm^−2^ which allows reaching temperatures upper than 1000°C at few minutes of exposure. Tests were performed at different periods of time that ranged between 5 and 90 min. The percentage of transformation of boehmite into alumina was followed by the water content of samples after solar radiation exposure. Samples were characterized by X-ray diffraction, infrared spectroscopy, and thermogravimetry. Metastable aluminas started to appear at 5 min and the crystalline and stable phase corundum at 10 min of solar radiation exposure.

## 1. Introduction

Alumina, the generic name for the aluminum oxide compound, is one of the most extensively materials used for a huge number of industrial applications (catalysts, ceramics, biomaterials, etc.) and its annual production in thousand metric tons exceeds 76300 [1]. Aluminas are mainly prepared by calcinations of precursors such as aluminum hydroxides and oxyhydroxides which can be in crystalline and gelatinous forms [2, 3]. The dehydration and/or dehydroxylation of precursors to form Al_2_O_3_ is an endothermic gas-solid reaction performed by a high temperature process with elevated energy consumption. Accordingly, the process costs are high from economical and environmental points of view.

In general terms, pollution should be considered as a global concern, and so the adoption of environmental-friendly measures to achieve the efficient use of natural resources and the management of industrial waste should be one of the main strategies for the sustainable development all over the world. Solar energy is an environmental friendly and renewable energy source which can be used to reduce the impact of energy coming from fossil sources. Although it is seasonal and geographically dependant, solar energy can be used for different applications, even those with high heat demand, by employing sunlight radiation concentrators [4, 5]. Solar radiation can be concentrated by refraction using a Fresnel lens. This type of concentrator has several advantages such as high optical efficiency with light weight, small volume, and low cost installation. Besides, Fresnel lens is nowadays made of polymeric material such as polymethylmethacrylate (PMMA) which exhibits high transmissivity for solar spectrum, thermal stability, and refraction index quite similar to glass devices, among others characteristics [6]. Concentrated solar energy (CSE) has been used for high temperature processes such as metal surface modifications [7, 8]. However, references about the direct application of CSE for reactions on powdered solids are scarce in literature [9, 10].

In a previous paper, a process was developed to obtain boehmite using a hazardous waste from aluminum industry as raw material [11] and the conversion of the as-obtained boehmite into *α*-alumina by calcinations in a muffle furnace at 1300 and 1400°C was studied [12].

Aluminum is the most exploited nonferrous metal with an increasing demand as the global population rise, due to its uses in transportation, building and construction, packaging, machinery and equipment, and so forth [13]. Therefore, the rising of the waste production and the continuous development should be performed in order to converge in a sustainable way. The huge number of researches carried out during the last decades focused on finding effective solution for the waste treatment has allowed some of these residues to become new raw materials for many industries. This achievement ensures saving energy consumption and natural resources, reducing the negative environmental impact and also encouraging the creation of secondary and tertiary industries [14, 15].

The use of concentrated solar energy is envisioned as a profitable energy source for the treatment of materials and wastes, and thus the aim of this paper is to study the transformation of boehmite obtained from a waste, into an added value material as alumina, by means of the thermal energy provided by sunlight. The as-obtained aluminas were characterized by X-ray diffraction (XRD), X-ray fluorescence (XRF), and infrared spectroscopy (FTIR). The percentage of transformation of boehmite according to solar radiation exposure time was determined by thermogravimetric analysis.

## 2. Materials and Methods

### 2.1. Materials

A sample of the alumina precursor, boehmite, was obtained by a sol-gel process using an aluminum waste as raw material following the method previously reported [12]. The aluminium waste, coming from the fine suction system used in the aluminium slag (dross) milling operation and supplied by a tertiary aluminium industry (Recuperaciones y Reciclajes Roman S.L., Fuenlabrada, Madrid, Spain), is a very fine grey coloured powder, with a characteristic odour derived from its aluminium nitride, carbide, and sulphide contents. The major mineralogical composition of the waste is as follows: 31.2% Al metal, 20.0% Al_2_O_3_ (corundum), 15.0% MgAl_2_O_4_ (spinel), 8.4% AlN, 8.0% SiO_2_ (quartz), 8.2% CaCO_3_ (calcite), 1.8% Fe_2_O_3_ (hematite), 1.5% TiO_2_, 1.5% chloride (Na/K), 0.7% Al_2_S_3_, and other minor metal oxides. The as-obtained boehmite (AlOOH·*n*H_2_O) is a white powdery solid composed mainly of 61.5% Al_2_O_3_ and 31.8% H_2_O, with minor components such as Fe_2_O_3_ and SiO_2_, among others [12].

### 2.2. Solar Energy Concentrator (SEC)

**Figure 1:**
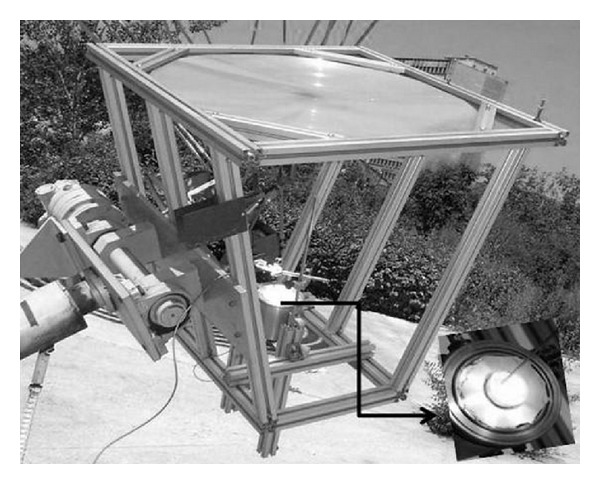
Solar energy concentrator (Fresnel lens) with a detail of the sample chamber and the positioned thermocouple.

Solar radiation was concentrated by means of a Fresnel lens made of a very high optical quality acrylic material (polymethylmethacrylate, PMMA). The lens is positioned on an aluminum installation which has a polar axis. The lens movement from east to west is controlled automatically by a computer and the solar height is hand positioned.  Figure 1 shows the solar installation (CENIM, CSIC) with a magnification of the sample chamber which is made of stainless steel and the thermocouple positioned into the sample crucible. The physical characteristics of the lens are collected in Table 1 [7, 9].

### 2.3. Experimental Procedure

**Figure 2:**
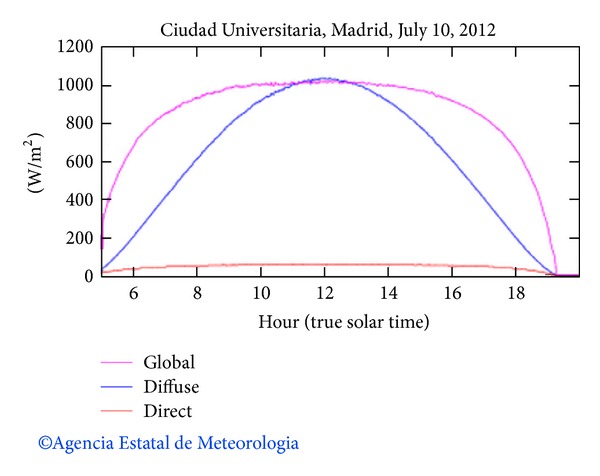
Local solar radiation curves according to AEMet (Spanish Agency of Meteorology http://www.aemet.es).

Samples of boehmite were placed into the sample chamber of the SEC (Figure 1) to the optimal focal distance. Crucibles of alumina and steel were used in order to study their effect on the transformation of boehmite into alumina. Alumina crucible, 4 cm height and 3 cm diameter, was loaded with 2.00 g of alumina precursor and the second one of 0.8 cm height and 1 cm diameter with 1.00 g of sample. Samples were slightly compacted to improve the heat transfer between grains. A type K thermocouple placed in the middle of sample was used to measure temperature. Tests were carried out to different solar radiation exposure times from 1 to 90 min, in summer, during the local time of the highest radiation (12.00 to 15.00 pm). An example of solar radiation (global, diffuse, and direct) during this season is shown in Figure 2. From hereinafter, samples are referred as *S*_*x*_, where *x* represents the exposure time to solar radiation in min.

### 2.4. Characterization Techniques

The chemical composition of samples was determined by X-ray fluorescence (XRF, PANalytical AXIOS wavelength-dispersive X-ray spectrometer) on compacted specimen of 37 mm diameter. X-ray diffraction (XRD) measurements for identification of crystalline phases were carried out using a Bruker D8 advance diffractometer with Cu K*α* radiation (1.541874 Å). Characterization of samples was completed by Fourier transform infrared (FTIR) spectroscopy (FTIR Nicolet Nexus 670–870); spectra were recorded on KBr compacted disk in the wavelength region of 1100–400 cm^−1^.

The transformation of boehmite into alumina was followed by the water content of sample after solar radiation exposure and determined by thermogravimetric analyses (TG) performed in a SDT-Q600, TA instrument at heating rate of 20°C·min^−1^, in air atmosphere up to 1200°C, using alumina crucibles with 20 mg samples.

## 3. Results and Discussion

**Figure 3:**
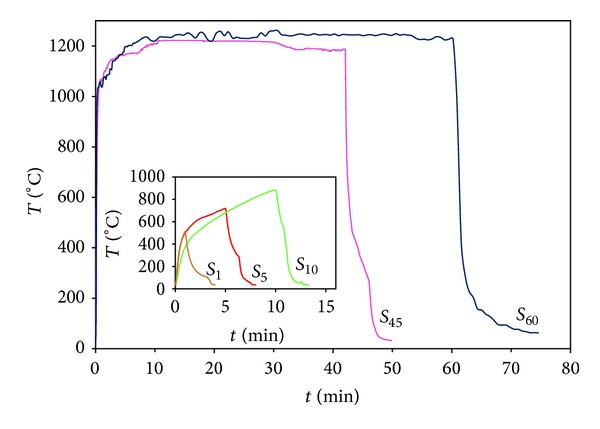
Curves of the temperature reached during different exposure times to solar radiation.

Figure 3 shows several curves of the temperature reached at different exposure times. As can be seen, values of temperature higher than 600°C were obtained at very short exposure time (lower than 1 min). In the case of long exposure time, high temperature (>1200°C) could be maintained for periods of time longer than 60 min (not shown in the figure).

**Figure 4:**
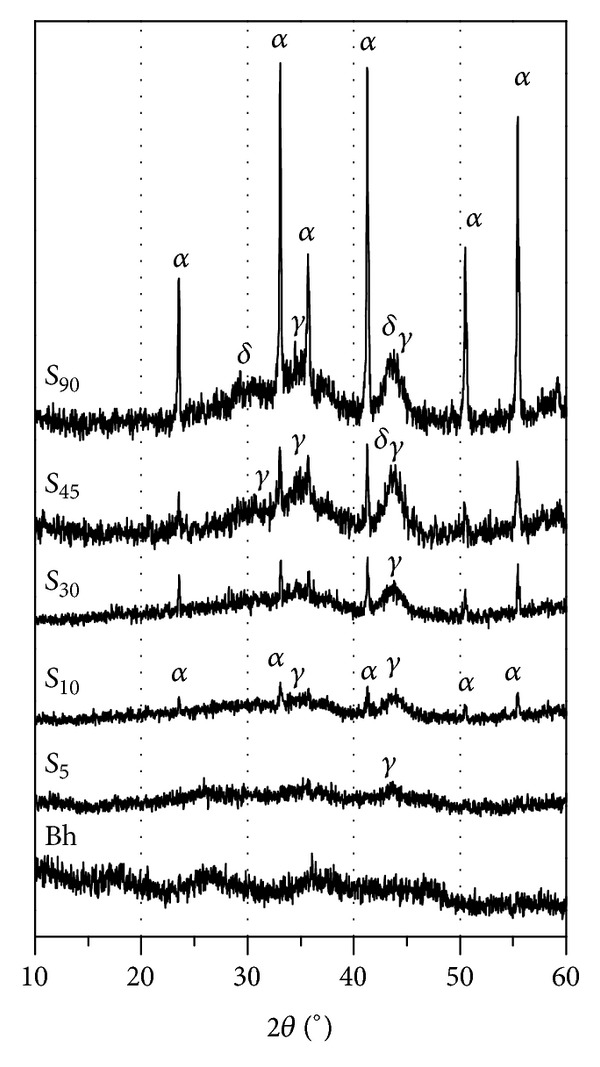
XRD patterns of boehmite (Bh) and sample obtained after 5 (*S*_5_), 10 (*S*_10_), 30 (*S*_30_), 45 (*S*_45_), and 90 (*S*_90_) min of solar radiation exposure.

XRD patterns of the precursor boehmite (Bh) and the samples obtained after solar radiation during different exposure times, using the alumina crucibles, are shown in Figure 4. XRD pattern of Bh exhibits a broad and diffuse profile which corresponds to a sample with very low crystallinity or very small crystallite size. The hardly observed maxima of diffraction peaks are centered approximately at 28.5, 38.1, and 48.5° 2*θ* and they can be attributable to the Bragg reflection on the planes (021), (130), and (002), respectively. The reflection on the (020) plane was not observed which is a characteristic of nonhydrothermally aged boehmite [16]. The aluminum content determined by XRF was 32.1 wt.% and the total mass loss determined by TG, which corresponds to the complete dehydroxylation/dehydration of boehmite, according to (1), was 39.1 wt.%, yielding a boehmite with the stoichiometry AlOOH·1.3H_2_O. Boehmite with variable water content is reported in literature depending on the synthesis process and the raw material among other experimental factors [12, 16]:

(1) $$\begin{array}{c} 2\text{AlOOH}\cdot {n\text{H}}_{\text{2}}\text{O}⟶{\text{Al}}_{\text{2}}{\text{O}}_{\text{3}}+\left(n+1\right){\text{H}}_{\text{2}}\text{O} \end{array}$$

After 5 min under concentrated sunlight exposure, a temperature of 700°C was achieved and pattern exhibits a XRD profile characteristic of samples with very disordered crystallographic structure, indicating that the transformation of boehmite into metastable alumina phases had took place [17, 18]. A small diffraction peak can be observed at 46.2° 2*θ* which could be attributable to the reflection of hkl index 400 corresponding to *γ*-Al_2_O_3_ (JCPDS 29-0063). Polymorphic phase transformation from boehmite into the metastable *γ* alumina is reported to occur in a wide temperature range according to the characteristic of the precursor [19]. At 10 min of solar radiation exposure, temperature was nearly 900°C, and the XRD pattern of sample (*S*_10_) shows diffraction peaks assigned to the hkl reflections reported in the JCPDS file 1-089-7717 for the *α*-Al_2_O_3_ (corundum), along with the previous one corresponding to *γ*-Al_2_O_3_. As the exposure time increases, temperature rises and it causes grain growth and better crystallinity of alumina phases. Other metastable polymorphs such as *δ*-Al_2_O_3_ can be formed when temperature is higher than 800°C [18]. After 15 min of solar exposure, a temperature value of 1200°C was attained and it could be maintained for several hours, depending on the local solar radiation (Figure 2). The longer exposure time, the better crystallization of alumina phases. Thus when time is double (from sample *S*_45_ to sample *S*_90_), the intensity of diffraction peaks increases.

**Figure 5:**
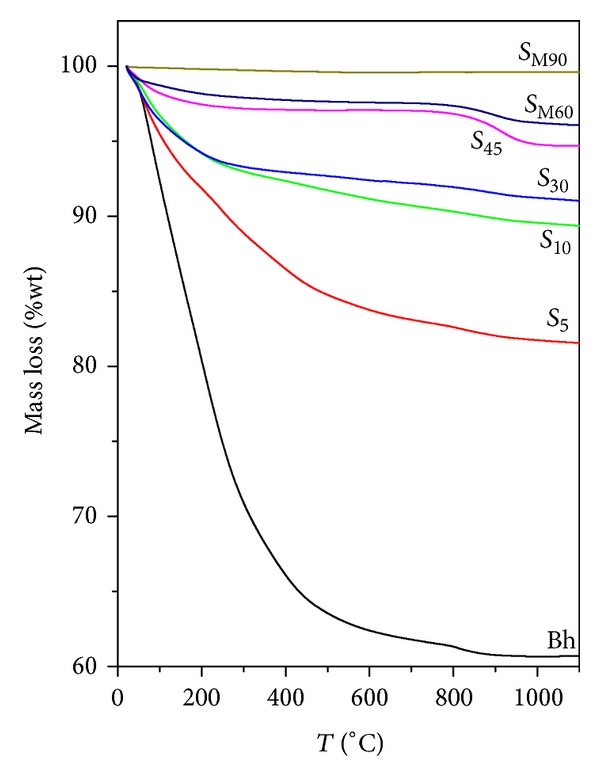
TG curves of initial boehmite and sample obtained after 5–90 min of solar radiation exposure (for samples *S*_M60_ and *S*_M90_, metallic crucible was used).

As the structural changes involved in the transformations boehmite-metastable aluminas-corundum occur very gradually, they will proceed continuously as shown in the TG curve of boehmite (Figure 5), leading to the coexistence of several alumina polymorphs. The coexistence of these phases is also favored for the high heating rate and for the short calcination time (shorter than two hours).

**Figure 6:**
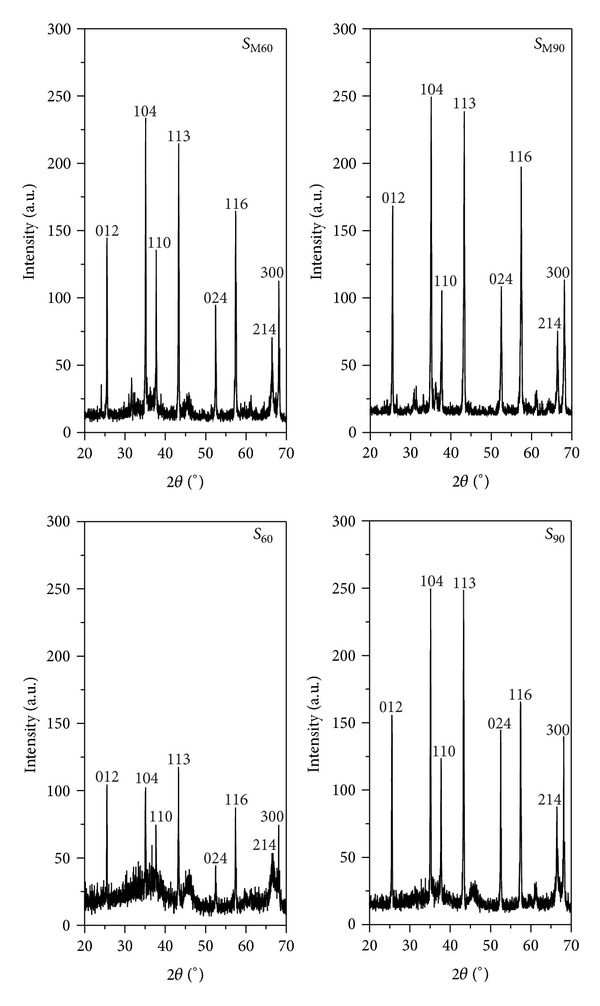
XRD patterns of samples after 60 and 90 min of solar radiation exposure, employed ceramic and metallic crucibles.

Figure 6 shows a comparison between XRD patterns of samples obtained after 60 and 90 min of solar radiation exposure, when ceramic and metallic crucibles were employed. For the same value of solar radiation exposure time, it is observed that the diffraction peaks intensity of *α*-Al_2_O_3_ increases and the width for all of them decreases when metallic crucible was used. This pattern corresponds to a highly crystalline material with narrow and well-defined diffraction peaks, which are undoubtedly assignable to the hkl reflections included in the JCPDS file 1-089-7717 of *α*-Al_2_O_3_ (corundum). Moreover, the significance of the background profile is much lower in this sample, indicating a higher transformation level of the bulk boehmite into corundum. Although the thermal conductivity is much higher in metallic crucible than that in ceramic one, this finding is nevertheless attributable to the smaller diameter of metallic crucible (10 mm) which is quite similar to the focus diameter of SEC and also to the lower temperature gradient from the surface to the bottom of the crucible because in this case the sample amount (height of the cylinder) is smaller and the complete surface of the sample receives the concentrated radiation.

**Figure 7:**
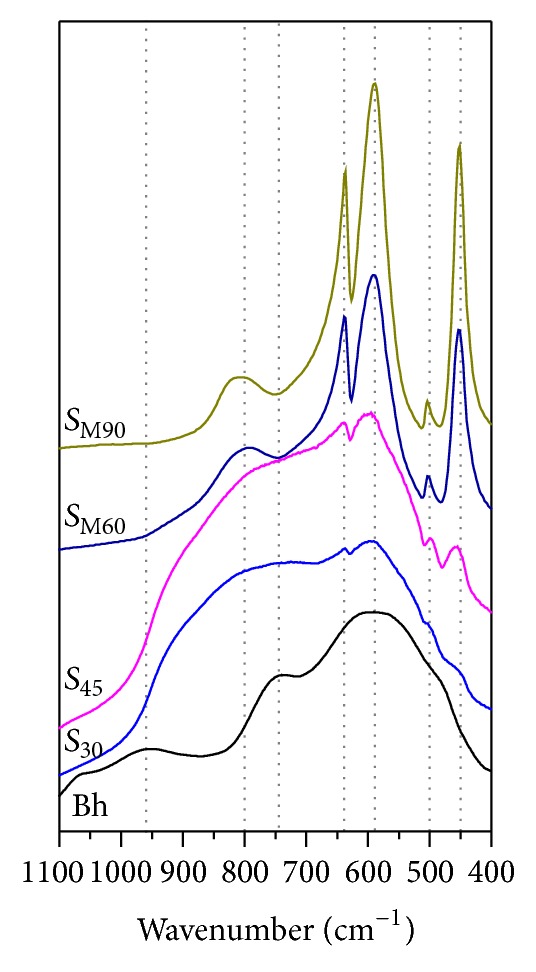
FTIR spectra of boehmite and samples after 30, 45, 60, and 90 min of solar radiation exposure.

Figure 7 collects the FTIR spectra of initial boehmite and samples after 30, 45, 60, and 90 min of solar radiation exposure, recorded in the wavenumber range from 1100 to 400 cm^−1^. Boehmite spectrum exhibits very broad bands centered at 481, 586, and 742 cm^−1^ corresponding to fundamental modes of vibration of amorphous or nanocrystalline for bulk crystal boehmite [20], in accordance with XRD results. The evolution of spectra with the solar radiation exposure is observed at low periods of time, and thus the bands corresponding to bending and stretching modes of Al–O bonds in the AlO_6_ group for the rhombohedral *α*-Al_2_O_3_ are perceptible for sample obtained at 30 min of solar energy exposure. The broadness of these bands indicates the presence of transitional aluminas. For sample at 90 min, these bands are well defined and narrow and they are centered at 451, 588, and 636 cm^−1^ indicating a well-defined structure of the corundum [12, 21]. IR spectroscopy results are therefore coherent with the evolution of the phases observed by XRD.

**Figure 8:**
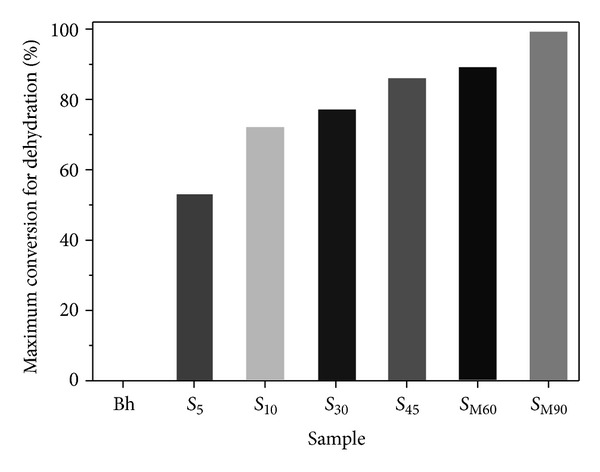
Percentage of transformation of boehmite into alumina with solar radiation exposure time.

The percentage of transformation of boehmite into alumina was determined by the water content of samples after solar radiation exposure. TG curves of samples after calcinations (Figure 5) show that water content decreases with exposure time. Taking into account the total water content of initial boehmite and (1), the results of the calculated percentage of transformation can be seen in Figure 8. A transformation higher than 50% was achieved at only 5 min of exposure. In spite of the fact that the sample is white and accordingly the absorption of solar radiation is not favored, a high percentage of transformation (>75%) is attained at 10 min of solar radiation exposure. A complete transformation was attained at longer exposure time (90 min).

## 4. Conclusions

The use of a solar energy concentrator such as a Fresnel lens allowed reaching high temperature (>600°C) at very short exposure time (lower than 1 min). Besides, high temperature (<1200°C) can be maintained for long time (several hours). This is highly attractive due to its characteristics of environmentally friendly and low cost. For conventional energy powered furnaces, the heating rate is lower, and accordingly the working temperatures are reached after longer time, being the cost of energy consumption high.

The feasibility of the used of concentrated thermal solar energy for providing the energy necessary to endothermic solid-gas reaction such as the transformation of boehmite into alumina was demonstrated. In our experimental design, the complete dehydratation of boehmite was attained after 90 min of solar radiation exposure and a well-crystallized corundum was obtained. For lower exposure time, other alumina polymorphs as gamma were obtained.

An upscaled installation and a better design of the sample chamber will improve the absorption of solar radiation.

## Figures and Tables

**Table 1:** Fresnel lens specifications.

Diameter (mm)	889
Surface (m^2^)	0.8
Thickness (mm)	3.17
Grooves (mm)	50 in 25
Focal distance (mm)	757
Circular focus diameter (mm)	8
Power density (W·cm^−2^)	260
Solar radiation concentration	2644 times
Refractive index	1.49
Transmission (%)	92
